# “BLUE BOARD” – an innovative knowledge management programme for educating health care practioners using blended learning modules

**DOI:** 10.1186/1753-6561-5-S6-P32

**Published:** 2011-06-29

**Authors:** CR Parathasarathy

**Affiliations:** 1 Microbiology, Chennai Medical College Hospital & Research Centre, Trichy, India

## Introduction / objectives

Existing infection control(IC) programs in hospitals are not effective due to lack of coordinated educational modules for stake holders in health care environment. We highlight our experience on a new knowledge management program which aims to educate and update information regarding IC practices to all Health Care Practitioners (HCP).

## Methods

A working core group was formed comprising of Heads of all clinical departments and Microbiology. Three members of the core group initiated a program at departmental level to identify their needs & issues, and to find out areas of constraints & possible remedial measures. The self generated team (BLUE BOARD) interacted through electronic media. A common platform was created so that different units could revise existing policies considering guidelines & feasibility of implementing the revised ones. Achievements were assessed by indicators viz., infection rate and antimicrobial usage. Data was analysed by simple descriptive statistics.

## Results

They met at regular intervals & utilized learning materials, guidelines & other scientific data from authorized resources. They contributed to revise the program based on feedback from members. This continuous on-going education cum training programme blended with computer technology, interactive discussions & role plays brought down infection rates (25%) and reduction in the use of antimicrobials (17%).

## Conclusion

“Blue Board” has streamlined IC practices, improved the attitude of HCPs. Based on the confidence gained, BLUE BOARD system extended their services to other hospitals within the locality, disseminated knowledge gained and maintained uniformity in IC practices.

## Disclosure of interest

None declared.

